# All-optical polarization control in time-varying low-index films via plasma symmetry breaking

**DOI:** 10.1038/s41566-026-01886-3

**Published:** 2026-04-03

**Authors:** Wallace Jaffray, Sven Stengel, Alexandra Boltasseva, Vladimir M. Shalaev, Maria Antonietta Vincenti, Domenico de Ceglia, Michael Scalora, Carlo Rizza, Marcello Ferrera

**Affiliations:** 1 Institute of Photonics and Quantum Sciences, Heriot-Watt University, Edinburgh, UK; 2 Elmore Family School of Electrical Computer Engineering and Birck Nanotechnology Center, Purdue University, West Lafayette, IN USA; 3 Department of Information Engineering, University of Brescia, Brescia, Italy; 4 Charles M. Bowden Research Center, Redstone Arsenal, Huntsville, AL USA; 5 Department of Physical and Chemical Sciences, University of L’Aquila, L’Aquila, Italy

**Keywords:** Ultrafast photonics, Optical materials and structures, Integrated optics

## Abstract

Controlling the polarization state of light with sub-picosecond speed and sub-wavelength precision remains a key challenge for next-generation nanophotonic devices. Conventional methods, such as birefringent crystals, liquid crystals or electro-optic Pockels cells are limited in speed, compactness and energy consumption. While structured materials and two-dimensional heterostructures show some promise for on-chip ultrafast performance, all-optical control at the nanoscale remains an open issue. Here we introduce an all-optical scheme that uses femtosecond pumping of low-index, sub-wavelength isotropic films to achieve ultrafast control over birefringence, dichroism and optical activity within a single-material platform. When the material is probed at its crossover wavelength, linearly polarized pumping induces a transient phase retardation between opportune orthogonal components as large as 0.1π μm^−1^, accompanied by a dichroic absorption ratio of ~1.2. When, instead, circularly polarized excitation is employed, the probe experiences non-reciprocal optical activity, leading to polarization rotation reaching 1.1° μm^−1^. These transient values are orders of magnitude larger than what is recorded from alternative nanophotonic systems and can be quantitively reproduced by a specialized model, which highlights the critical role of time-varying damping in photoexcited carrier plasma. Our combined experimental and theoretical study establishes a reconfigurable, deep-sub-wavelength polarization-control mechanism operating on sub-picosecond timescales. This approach is ideally suited for compact ultrafast modulators, dynamic metasurfaces and tunable non-reciprocal photonic devices, with broad implications for quantum optics, ultrafast logic and time-resolved sensing.

## Abstract (web summary)

Femtosecond pumping of an isotropic low-index subwavelength film enables ultrafast polarization control, with induced birefringence up to 0.1π μm^−1^, a dichroic ratio of ~23% and a non-reciprocal optical activity of ±1.1° μm^−1^.

## Main

Controlling the polarization state of light with high speed, precision and reconfigurability is of foundational importance across many fields of modern photonics and optical technologies. Polarization-sensitive functionalities enable a broad range of applications, from classical and quantum communication to ultrafast optical computing, sensing and advanced microscopy^1–3^. Integrated polarization control is particularly vital in planar or on-chip photonic architectures, where conventional bulk elements are unfeasible due to their size, tuning limitations or speed bottlenecks^4^.

Traditional systems such as static birefringent crystals, liquid crystals and electro-optic Pockels cells enable polarization transformation but are inherently constrained by slow response times, poor miniaturization potential and lack of active programmability^5–7^. To address these limitations, substantial effort has been directed at emerging platforms such as structured metasurfaces and van der Waals (two-dimensional) heterostructures, to achieve compact and fast polarization control^8–13^. Metasurfaces, for instance, have shown strong static anisotropy and engineered dichroism through spatial patterning^3,14–17^, whereas two-dimensional materials and heterostructures offer strong nonlinearities and quantum confinement effects suitable for polarization-dependent interactions^18,19^. However, these devices remain limited to a single polarization mechanism (typically dichroism or birefringence), which is preset by fabrication and often relies upon resonant elements such as plasmonic nanoantennas or dielectric micro-resonators^20–27^. These resonant dependencies impose constraints on speed and spectral bandwidth, hindering their use in practical ultrafast systems, whereas static structures do not allow for dynamic reconfigurability. Regarding fully integrated polarization control, results have been attained from highly nonlinear systems such as colloidal nanoparticles^28^ and ultrathin metallic films^29^. Specifically, these systems have been targeting the inverse Faraday effect (IFE)^30–33^ to optically induce chiral properties by triggering a magnetic field and achieving optical activity of the order of 10^−5^–10^−4^° μm^−1^. Recently, epsilon-near-zero (ENZ)-based resonant architectures have been used for the design of anisotropic perfect absorbers^34^. In this work, multi-layer resonant structures are used to attain a reflective polarizer with a polarization extinction ratio of 91 with an ON/OFF time of about 800 fs. However, these structures are limited in bandwidth, operate only on dichroism, lack tunability and require stringent excitation parameters due to the reliance upon Berreman modes. Within the context of ultrafast polarization manipulation, we should also mention ref. ^35^. In their work, a Au-grating/ITO multi-layer is used to induce a 19° pump-dependent polarization rotation over the main axes of the elliptical transmitted signal. However, the unpumped structure is strongly anisotropic and birefringent, with a pronounced wavelength dependence between s and p polarization components. Additionally, it does not enable tunable gyrotropic behaviour, whereas the metallic nano-structuring lowers the damage threshold and increases fabrication complexity.

**Fig. 1:**
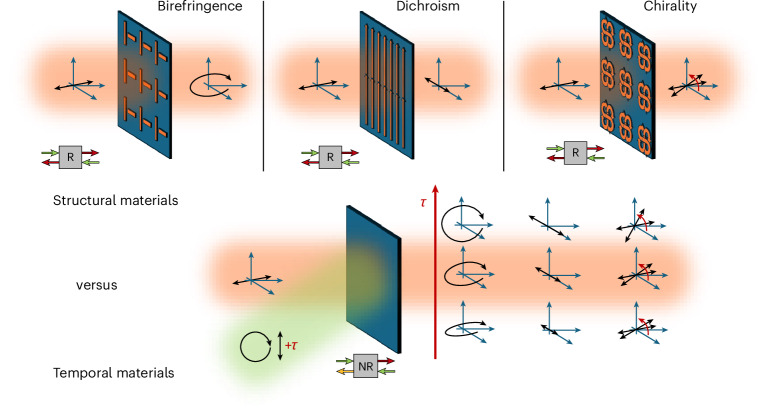
Conceptual comparison between structural and temporal approaches to polarization control. Top: structural metasurfaces employ geometrically distinct designs to achieve reciprocal (R) birefringence, dichroism or chirality, each requiring separate fabrication. Bottom: in contrast, temporal materials respond to tailored optical excitations, where the time profile and polarization of the incident beam dynamically induce birefringent, dichroic or chiral behaviours in a single unpatterned substrate. This temporal engineering approach greatly enhances reconfigurability, reduces fabrication overhead and expands the accessible optical response space while also enabling non-reciprocal functionalities.

A new photonic framework has recently emerged from time-varying photonic systems, enabled by ultrafast optical nonlinearities in low-index transparent conducting oxides (TCOs)^36–39^. Within this context, polarization control over all fundamental transformations (birefringence, dichroism and gyrotropic effects) has been discussed in theoretical contexts^40–44^, whereas the experimental literature solely pertains to scalar nonlinearities. These isotropic nonlinearities preserve the material symmetry and do not generate the required tensorial response for controlling birefringence, dichroism and/or optical activity. The emergence of time-varying systems marks a fundamental shift in how optical functionalities can be engineered^45^. Rather than relying solely on static, lithographically defined structures to control polarization, time can be used as an additional degree of freedom. This transition redefines the paradigm of photonic design, expanding the operational bandwidth and adaptability of optical systems by transferring tailorability from nanofabrication steps towards the shaping of the optical excitation. Figure 1 clearly illustrates this distinction between the structural and the temporal approaches to polarization control via flat systems. Structural metasurfaces require a separate geometry to realize birefringence, dichroism or chirality, and their response is fixed and reciprocal, as shown in the top row of Fig. 1. In contrast, a temporal approach allows a single unpatterned medium to perform all these tasks by shaping the excitation pulse, thus enabling functional switching, dynamic tuning and non-reciprocal behaviour, as shown in the bottom row of Fig. 1 (ref. ^46^).

Fundamentally, two different targets exist in advanced optical wavefront control: tunability and reconfigurability. Tunability refers to the modulation of a given parameter within a fixed functionality. Conversely, reconfigurability denotes the ability of a system to dynamically switch between distinct tasks. Our all-optical, time-varying platform addresses both these targets simultaneously. By pumping an unpatterned and isotropic, low-index thin film of aluminium zinc oxide (AZO), we achieve ultrafast tunability of the complex refractive index and dynamic reconfigurability of the induced anisotropy, all on sub-picosecond timescales. This enables the same film to behave sequentially as birefringent, dichroic or with optical activity, depending on the pump conditions.

More specific to the state of the art, conventional metasurfaces, although providing high spatial resolution through static patterning, have limited tunability and no true reconfigurability. Their tunability relies upon relatively slow electro-optic, thermal, optomechanical or phase-change mechanisms^47,48^. In contrast, spatial light modulators offer full reconfigurability but are limited by ~10-μm pixel size, ~1-kHz refresh rate and low damage thresholds. Our time-varying transparent-conductor films combine the strengths of both approaches. Their spatial resolution is ultimately limited only by diffraction, whereas the nonlinear bandwidth of AZO spans over 260 nm (ref. ^49^). The optical pump can also be spatially structured, to enable global response via local vectorial control. This would establish an ultrafast reconfigurable flat-optical platform that functions as an optical-frequency analogue of a high-resolution femtosecond-fast nonlinear spatial light modulator.

At the core of our experiments lie sub-wavelength transparent conducting oxide films of AZO operated near their ENZ point. Using linearly and circularly polarized pump beams at variable delays from a weak probe, we demonstrate all-optical control over the three fundamental polarization interactions, namely, birefringence, dichroism and optical activity. All this has been achieved on sub-picosecond timescales. Under linearly polarized pumping, we report induced birefringence Δ*φ*_L_ ≈ 0.1π μm^−1^ (here defined as nonlinear phase retardation per unit length between orthogonal polarization components) and a dichroic absorption ratio Δ(*P*_*x*_/*P*_*y*_) ≈ 23% (where *P*_*x*_ and *P*_*y*_ are the measured probe transmitted powers along the orthogonal reference axes). Subsequently, under circularly polarized excitation, we observed induced optical activity of ±1.1° μm^−1^ for a linearly polarized probe. This induced polarization rotation was measured to be clockwise or counter-clockwise depending on the pump helicity. These results experimentally realize key concepts that were previously proposed in theoretical frameworks, such as dynamic chiral modulation in time-varying media^50,51^. In absolute terms, these numbers correspond to 0.09π for birefringence and ±1° for optical activity in the 900-nm film.

Critically, our system achieves polarization reconfigurability over a broad operational bandwidth with extreme temporal agility. More specifically, the nonlinear response in AZO films is dominated by free-electron-driven metallic nonlinearities, whose characteristic resonance is the plasma frequency. Unlike Lorentz-type resonances, the plasma response is intrinsically broadband, resulting in a wide operational spectral range able to accommodate ultrafast optical pulses down to the few-cycle regime^49,52^. We note that the observed behaviour is not influenced by geometrical (Fabry–Pérot-like) or plasmonic (surface-mode) resonances. The former is suppressed by the deep-sub-wavelength AZO thicknesses and ENZ losses, whereas the latter cannot be excited as surface-bound plasmonic/ENZ modes lie outside the light cone and are not phase-matched under our near-normal incidence (low numerical aperture) excitation.

To interpret these phenomena, we develop an effective model that captures the temporal evolution of the material’s anisotropic electron distribution and resulting dielectric tensor asymmetry. This model qualitatively reproduces all observed polarimetric responses and links the symmetry-breaking mechanism to ultrafast non-equilibrium carrier dynamics. This feature is uniquely enabled by the absence of geometric anisotropy.

Here we demonstrate simultaneous access to all three major polarization control mechanisms on a single-material platform, achieved purely through temporal engineering of the pump field. This decoupling of spatial fabrication and temporal functionality introduces a new paradigm for reconfigurable nanophotonics and opens pathways to ultrafast polarization modulators, dynamic metasurfaces, optically addressable non-reciprocal devices and ultrafast computing in both classic and quantum regimes^53–55^.

## Experimental settings

Experiments were conducted using a pump–probe set-up designed to investigate the ultrafast modulation of polarization states in a time-varying, sub-wavelength film of transparent conducting oxides. The sample used was a 900-nm-thick AZO film deposited via pulsed laser deposition onto a fused silica substrate in a low-oxygen environment^45,56^. The linear optical response of the unpumped film is isotropic, making it ideal for isolating nonlinear time-varying polarization effects. The probe beam was supplied by an optical parametric amplifier, which provided pulses with output durations of 85 fs, centred at 1,250 nm, near the ENZ crossover wavelength of the AZO film. The pump beam was fixed at 787 nm with a pulse duration of 100 fs, a repetition rate of 10 Hz and a peak intensity of approximately 800 GW cm^−2^. Under these conditions, thermal build-up is negligible and pump intensity is well below the reported damage threshold for AZO films (measured to be a few terawatts per centimetre squared at 1 kHz)^49^. While the pump beam was set exactly at normal incidence, the probe was set at a small (<5°) incidence angle. This configuration allows for spatial separation between probe and pump, allowing for an optimal signal-to-noise ratio. Temporal overlap was achieved by recording nonlinear changes of the probe as the delay was varied in steps of 10 fs. Initially, the pump was polarized linearly along 0° (horizontal and aligned with the plane of incidence), with a beam diameter of ~1.5 mm (1/*e*^2^), considerably larger than the linearly polarized probe beam diameter (~200 μm), ensuring uniform excitation of the probed region. The probe intensity was set over three orders of magnitude below the pump intensity to avoid self-action nonlinearities. Additional information, including fabrication, verification of sample’s linear isotropic behaviour, frequency-resolved optical gating measurements of probe pulse, material dispersion curves and thermal analysis, are all available in the Supplementary Information.

**Fig. 2:**
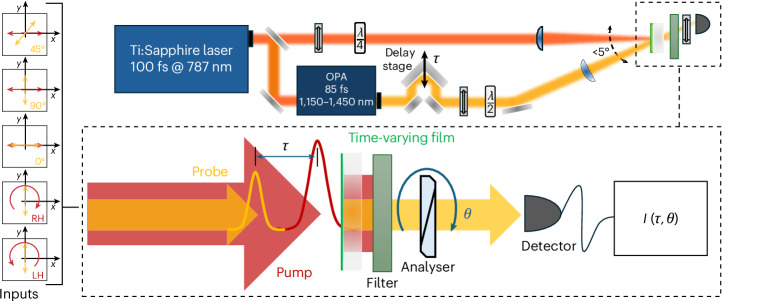
Experimental settings. A time-varying sub-wavelength thin film is attained by optically pumping a 900-nm aluminium zinc oxide (AZO) layer via a 10-Hz train of 100-fs pulses centred at 787 nm. The time-varying film is probed with pulses (85 fs) centred at 1,250 nm, which is within the ENZ bandwidth of the material. Panels along the left side explain the various input experimental configurations of pump and probe polarizations, where RH and LH denote right-handed and left-handed polarization statuses, respectively. The output intensity *I* is measured as a function of both the pump–probe delay *τ* and the angle *θ* between the input polarization and the optical axis of the output analyser. The upper section outlines the experimental set-up where the polarizers and phase retarders used in this study are indicated.

Polarization-resolved measurements of the transmitted probe were obtained by placing a motorized analyser along the transmitted beam path, after a frequency filter dedicated to remove any residual pump, and then measuring the output power at discrete angular steps of 2°. All data were averaged over multiple laser shots to improve the signal-to-noise ratio. For a first set of measurements pertaining to optically induced birefringence and dichroism, we used three input probe polarization states: 0°, 45° and 90° (that is, horizontal, diagonal and vertical with respect to the horizontal pump polarization), while the pump-to-probe delay *τ* was swept in steps of about 10 fs (Fig. 2). Subsequently, to investigate the induced optical activity, the probe was vertically polarized at 90°, while the pump was alternatively arranged into right circularly polarized (RCP) and left circularly polarized (LCP) states (notice that throughout the paper we assume for all polarization diagrams that light propagates inwards towards the display plane). All these configurations are reported in Fig. 2, together with all used inputs, which are described by associated icons.

The observed polarization transformations were analysed in terms of induced birefringence (phase retardation under linear pumping), dichroism (differential absorption under linear pumping) and optical activity (polarization rotation under circular pumping). These are evaluated from the angle-and-time-dependant probe intensity *I*_exp_(*τ*, *θ*) as recorded at the detector located in front of the analyser.

## Results and discussion

### Transient birefringence and dichroism

**Fig. 3:**
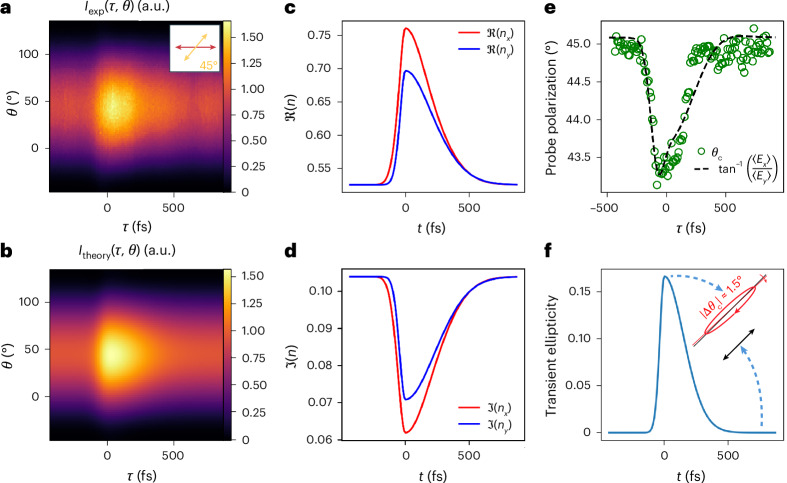
Ultrafast nonlinear polarization coupling in a time-varying AZO film. Experimentally measured and theoretically reconstructed polarization-resolved probe transmission through time-varying, 900-nm-thick aluminium zinc oxide (AZO) layer. **a**, Raw experimental intensity ${I}_{\exp }(\tau ,\theta )$ as a function of analyser angle *θ* and pump–probe delay *τ*, with the probe polarized at 45° and the pump at 0°. **b**, Simulated intensity map based on fitted complex indices, accurately reproducing the measured transmission with root mean square error below a few per cent. **c**, Real part of the transient refractive index retrieved from fitting the measured intensity, showing anisotropic changes along the *x* (red) and *y* (blue) directions. **d**, Imaginary part of the refractive index as retrieved from fitting, showing transient, polarization-dependent absorption. **e**, Extracted polarization angle of the transmitted probe from both experimental centroid analysis (green circles) and model (black dashed line), demonstrating excellent agreement. The theoretical evaluation is attained by using the formula reported in the inset, where the angled brackets indicate a time average integral over absolute time *t*. **f**, Retrieved ellipticity of the transmitted pulse as a function of time. The plot indicates induced nonlinear birefringence, whereas the inset shows the reconstructed polarization ellipse (at scale) for maximum birefringence, with ellipticity approaching a 1:7 ratio. The case for the unpumped polarization is also reported by the black bi-directional arrow (length is set to scale with the transmitted intensity).

With the probe polarization at 45° and the pump polarization at 0° (parallel to the plane of incidence), we can recover both the induced transient birefringence and dichroism. Figure 3a provides the raw experimental data for this case, where ${I}_{\exp }(\tau ,\theta )$ is the recorded probe intensity as a function of pump–probe delay *τ* and analyser angle *θ*. At early delays, in the absence of pumping, the probe maintains its linearly polarized state after transmission, and the analyser scan leads to a symmetric cosine-squared profile with no angular shift, as expected from Malus’s Law. At subsequent delays, where the probe is overlapped with the pump, there is an increase in transmission and a shift in the centroid of ${I}_{\exp }(\tau ,\theta )$. To further analyse this process we can mathematically represent the measured intensity after both the time-varying film and the analyser as follows:

1 $$\begin{array}{rcl}{I}_{\exp }(\tau ,\theta )&=&{\displaystyle\int}_{-\infty }^{\infty}\left[{\cos }^{2}\theta |{E}_{x}(t,\tau ){|}^{2}+{\sin }^{2}\theta |{E}_{y}(t,\tau ){|}^{2}\right.\\&& \left.+2|{E}_{x}(t,\tau )||{E}_{y}(t,\tau )|\sin \theta \cos \theta \cos \delta (t,\tau )\right]{\rm{d}}t,\end{array}$$

where *E*_*x*_(*t*, *τ*) and *E*_*y*_(*t*, *τ*), are the instantaneous, transmitted complex electric fields in the *x* direction (0°) and *y* direction (90°), respectively, as a function of absolute time *t* and for a given pump–probe delay *τ*. Here, *δ*(*t*, *τ*) is the phase difference between *E*_*x*_ and *E*_*y*_, which relates to the transient birefringence of the material. The integral over time deals with the power acquisition over the pulse duration. As the temporal profile of our input probe pulse is known (evaluated via frequency-resolved optical gating measurements), we can fit equation (1) to the intensity heatmap in Fig. 3a to recover a transient complex index in the *x* and *y* directions. Figure 3b shows the fitted heatmap, which closely matches the experimentally acquired data with less than a few percent root mean square error. The real and imaginary components of the time-varying index are provided in Fig. 3c,d, respectively. Here, the red lines show the complex index as perceived by the horizontal field components of the probe, whereas the blue lines show the complex index in the *y* direction. The recovered temporal index profile is remarkably aligned with previous measurements of time-varying index in AZO^57^ (see Supplementary Appendix D for extra details on the retrieval process).

To further verify the accuracy of our experimental fit, and our understanding of the nonlinear process, we calculate the polarization angle of the transmitted light as a function of the pump–probe delay. This is done using the previously evaluated complex index (Fig. 3c,d) and a temporal average over the absolute time *t* (shown as the black dotted line in Fig. 3e). This is then compared to the centroid of the experimental data shown as the green circles in Fig. 3e. This provides us with a direct estimate of the polarization angle of the transmitted light, which matches closely with the theoretical treatment (see ‘Material model’ section). Additionally, it is worth noting that the polarization of the transmitted light is rotated towards the horizontal pump by 1.5° after propagating only 900 nm. We can also recover the transient birefringence of the film, which imparts ellipticity to the transmitted pulses as shown in Fig. 3f. Specifically, as the probe interacts with the time-varying film, it gains a substantial ellipticity up to a ratio of almost 1:7 due to the induced difference between orthogonal index components (see to-scale ellipse in inset of Fig. 3f)^58^.

**Fig. 4:**
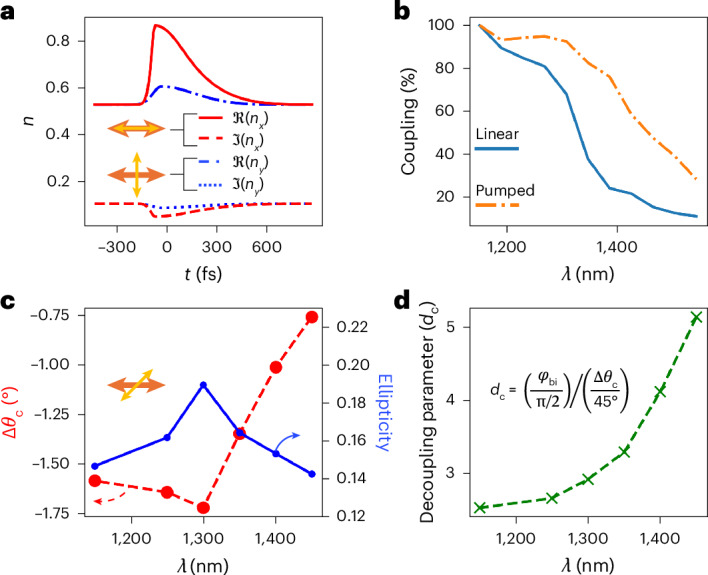
Extended analysis of dichroism and birefringence as a function of probe polarization and wavelength. **a**, Extracted transient complex index for a probe polarized in the *x* direction (red curves) and in the *y* direction (blue curves) for a horizontally polarized pump (see descriptive icons). **b**, Power coupling efficiency into the 900-nm AZO film against wavelength for pumped (orange dotted-dashed line) and unpumped (blue solid line) cases. **c**, Imparted probe polarization rotation (Δ*θ*_c_) and ellipticity as a function of probe wavelengths at maximum temporal overlap. Probe polarization is set at 45° with respect to the horizontal pump, as shown in descriptive icon. **d**, Decoupling parameter between dichroism and birefringence for different probe wavelengths. Formula for decoupling parameter *d*_c_ is shown in inset with *φ*_bi_ being the nonlinear birefringence phase shift. At wavelengths above the film’s ENZ crossover, the maximum achievable dichroism diminishes much faster than the birefringence, allowing these two effects to be partially decoupled.

Figure 4a illustrates the cases of a vertically polarized probe and a horizontally polarized probe, both for a horizontally polarized pump excitation. As previously described, we use our model to accurately retrieve the complex refractive indices along the specific direction of the probe polarization (see Supplementary Appendix E.2 for full experimental results). For the sake of absolute precision, we note that when comparing the refractive-index curves in Fig. 3c,d with those reported in Fig. 4a, small discrepancies appear. However, these differences lie very close to the uncertainty imposed by experimental limitations and can be ascribed to the dependence of the signal-to-noise ratio with the probe polarization angle. Importantly, these minor discrepancies do not affect the interpretation of the results.

The dominant nonlinear mechanism in the studied AZO films is the hot electron effect, which modifies the effective mass of the conduction electrons effectively forming an ultrafast light-driven plasma^59^. Under optical excitation the pump increases the electron momentum along its polarization direction, driving electrons up into the conduction band. These hot carriers subsequently scatter, transferring energy to the lattice (electron–phonon scattering) and to other electrons (electron–electron scattering), with the material quickly returning to its linear optical state. Crucially, electron–electron scattering redistributes the momentum imparted by the pump to off-axis directions. This alters the off-axis effective carrier mass and consequently the complex refractive index in those directions. The angular dependence of this scattering process is governed by a Yukawa-screened Coulomb potential, which is consistent with our experimental observation^60,61^.

Although the reported core analysis refers to a probe near the ENZ crossover where nonlinear effects are maximized, it is worth investigating the wavelength dependence of our nonlinear polarization control capabilities. To start, we can consider the very practical problem of efficiently injecting light into our material. To this end, in Fig. 4b we report the coupling into the film (power transmission through the first interface) versus probe wavelength in both the linear (blue line) and pumped (dotted-dashed orange line) regimes. In the pumped case, the coupling efficiency into the film is dramatically increased, making our operational conditions more energy efficient.

It is also interesting to consider how the effects of birefringence and dichroism can be selectively emphasized or suppressed through appropriate wavelength tuning. In this regard, we should keep in mind that the Kramers–Kronig relations prevent complete disentanglement of phase and absorption responses within a generous spectral window. Near the ENZ crossover the material transitions from dielectric to metallic, and the balance between absorption-driven and phase-driven nonlinearities changes considerably^45^. This is demonstrated by the results reported in Fig. 4c, where maximum imparted probe polarization rotation (red dashed line) and ellipticity (blue solid line) are reported as a function of probe wavelength for a 45° polarized probe. These two effects are directly linked to birefringence and dichroism, respectively. We find that as the probe wavelength moves above the ENZ crossover, the dichroic response diminishes more rapidly than the birefringent phase shift. To quantify this behaviour, we introduce a dimensionless decoupling parameter, *d*_c_, defined as the ratio of the birefringent phase shift *φ*_bi_ (normalized to π/2, which corresponds to circular polarization) to the dichroic rotation Δ*θ*_c_ (normalized to the maximum rotation of 45°, required to align the probe with the pump polarization). Large values of *d*_c_ therefore indicate a regime where birefringence dominates over dichroism, whereas low values of *d*_c_ indicate the opposite. As shown in Fig. 4d, there is a marked increase in decoupling above the ENZ crossover, demonstrating that wavelength tuning can push the system into a regime where birefringence becomes by far the predominant nonlinear effect. This provides a viable route for additional nonlinear polarization control.

Given that birefringence is primarily a propagation effect, one may also be interested in checking how film thickness affects the decoupling parameter. In this regard, we measure the dichroism and birefringence from a 300-nm AZO film (with similar ENZ crossover to the 900-nm sample) under the same experimental conditions previously met for the 45° polarization case. In absolute terms, when moving from the 900-nm sample to the 300-nm sample, the measured absolute dichroism goes from 1.5° to 0.25°, whereas the birefringence goes from 0.09π to a value below the detectability threshold. This corresponds to a reduction in dichroism by a factor of 6 and a reduction in birefringence by at least 15 times (considering a conservative noise floor). This nonlinear scaling of polarization control properties with thickness can be ascribed to three fundamental facts: (1) transparent-conductor optical behaviour is well known to be thickness dependant^62^; (2) laboratory-scale fabrication methods typically exhibit lower run-to-run reproducibility than industrial manufacturing processes; and (3) intrinsically, nonlinear effects in lossless materials scale linearly with propagation distance (for example, self phase modulation); however, when material losses become non-negligible (as for our case), this is no longer valid. The full results of this measurement can be found in Supplementary Appendix E.1.

### Induced optical activity

To investigate induced optical activity, we replace the linearly polarized pump with circularly polarized excitation, while keeping the probe fixed in a vertically polarized state (90°). By switching between RCP and LCP pump, we examine the induced transient optical activity and its non-reciprocal properties in an otherwise isotropic system.

**Fig. 5:**
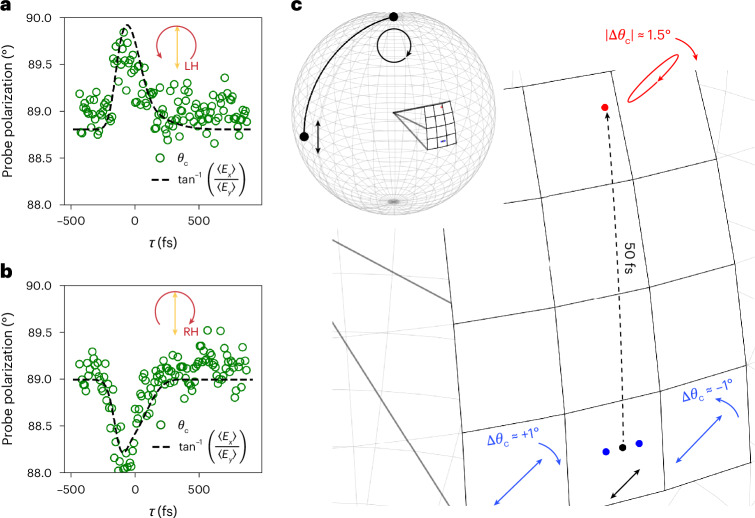
Pump-induced transient optical activity and overall polarization transformations on Poincaré sphere. **a**, Polarization rotation angle for an LCP pump, which is extracted from the experimental data (green circles) and from the theoretical fit (black dashed line). The theoretical evaluation is attained by using the formula reported in the legend, where the angled brackets indicate a time average integral over absolute time *t*. **b**, Polarization rotation angle for an RCP pump, which is extracted from the experimental data (green circles) and from the theoretical fit (black dashed line). As expected, the RCP-pumped nonlinearity has the opposite effect to the LCP-pumped case. The absolute rotation reaches a maximum value of approximately 1.1° μm^−1^ in both cases. **c**, Diagram of our nonlinear ultrafast polarization transformations on the Poincaré sphere. In the top left corner the inset shows the full Poincaré sphere to provide context and eliminate ambiguity on orientation of the sphere itself. The main image provides a zoom of our time-varying polarization transformations. Starting from a linear polarization (black bi-directional arrow), we show that a linearly polarized pump imparts both ellipticity and dichroism (see red ellipse icon). Meanwhile the left- and right-handed circular pumps rotate the linear probe polarization along the equator in opposing directions. Polarization states are reported up to scale, with major diagonal length indicating associated transmission. The dotted black line pertains the temporal dynamics of our polarization transformation under linear pumping.

To quantify the effect, we complete the same analysis as in the previous section (fitted intensity profiles and experimental data can be found in Supplementary Appendix E.2). The instantaneous polarization angle is provided in Fig. 5a,b, and is directly extracted from both ${I}_{\exp }(\theta ,\tau )$ (green circles) and from our fit (dashed black lines). Our theoretical evaluation of the instantaneous polarization angle is calculated using ${\tan }^{-1}(\frac{\langle {E}_{x}\rangle }{\langle {E}_{y}\rangle })$, where the angled brackets indicate a time average integral over the absolute time *t*. The polarization axis rotates clockwise for RCP and counter-clockwise for LCP, reaching absolute peak values of approximately ±1° within the duration of the pump–probe overlap. This corresponds to a non-reciprocal rotation rate of ~1.1° μm^−1^, which is several orders of magnitude higher than those achieved in other integrated systems^28,29^.

Unlike intrinsic material chirality, which is fixed in sign and magnitude, the response here is dynamically induced and tunable. The mechanism stems from an asymmetric redistribution of carriers in the presence of circularly polarized electric fields, which transiently breaks mirror symmetry in the electron plasma.

Finally, to provide a general overview of all triggered polarization transformations, these are reported on the Poincaré sphere space in Fig. 5c. Here, polarization states are drawn to scale with the major diagonal length proportional to the associated transmission. The black bi-directional arrow shows the initial linearly polarized probe state, and the red ellipse represents the polarization transformation under linear pumping. Finally, the case of induced optical activity under circular pumping is shown by blue bi-directional arrows.

### Material model

In our material model, the intrinsic time dependence of the effective mass modifies both the plasma frequency and the effective scattering rate^63^. Although the latter is often overlooked, it plays a decisive role in nonlinear polarization coupling. When the pump is circularly polarized, the time-varying mass term acquires the symmetry of the pump and reorganizes into a gyrotropic contribution that is algebraically equivalent to a Lorentz-like term acting on the probe. This correspondence does not imply that the IFE is a damping mechanism; rather, it shows that parametric modulation of the effective mass can generate both longitudinal (damping-like) and transverse (Lorentz-like) components. The IFE itself remains a nonlinear channel activated by circular polarization, while the ‘renormalization’ of the damping coefficient reflects how the pump-induced carrier mass modulation links to an emergent time-varying effective magnetic field **B**_eff_. For circularly polarized pumping, this framework clarifies why the transient behaviour of the effective damping parameter is inseparably linked to the emergence of the IFE^30–33^.

Returning to our specific experimental configuration, where the probe intensity is much lower than that of the pump, the dynamics of the probe can be represented by the following oscillator equation modified for a time-varying damping and plasma frequency:

2 $$\frac{{{\rm{\partial }}}^{2}{{\boldsymbol{\mathcal{P}}}}_{2}}{{\rm{\partial }}{t}^{2}}+{\overleftrightarrow{\gamma }}_{2}(z,t)\frac{{\rm{\partial }}{{\boldsymbol{\mathcal{P}}}}_{2}}{{\rm{\partial }}t}={\varepsilon }_{0}{\omega }_{{\rm{p}}}^{2}\,{\overleftrightarrow{F}}_{2}(z,t){{\boldsymbol{\mathcal{E}}}}_{2},$$

here ${\boldsymbol{\mathcal{P}}}_{2}(z,t)$ is the polarization vector driven by the probe field, ${\boldsymbol{\mathcal{E}}}_{2}(z,t)$ is the electric field of the probe, *ω*_p_ is the plasma frequency, *ϵ*_0_ is the vacuum permittivity, ${\overleftrightarrow{\gamma }}_{2}(z,t)$ is the time-varying scattering rate acting upon the probe and ${\overleftrightarrow{F}}_{2}(z,t)$ is a time-dependant modification factor accounting for changes in the plasma frequency. These latter two values are time-varying tensors and can be written as

3 $${\overleftrightarrow{\gamma }}_{2}={\gamma }_{1}\overleftrightarrow{I}+\alpha {F}_{1}\left[\begin{array}{cc}{{\mathcal{E}}}_{1,x}\frac{\partial \,{{\mathcal{P}}}_{1,x}}{\partial t} & {{\mathcal{E}}}_{1,y}\frac{\partial \,{{\mathcal{P}}}_{1,x}}{\partial t}\\ {{\mathcal{E}}}_{1,x}\frac{\partial \,{{\mathcal{P}}}_{1,y}}{\partial t} & {{\mathcal{E}}}_{1,y}\frac{\partial \,{{\mathcal{P}}}_{1,y}}{\partial t}\end{array}\right]$$

4 $${\overleftrightarrow{F}}_{2}={F}_{1}\overleftrightarrow{I}-\frac{\alpha {F}_{1}}{{\epsilon }_{0}{\omega }_{{\mathcal{P}}}^{2}}\left[\begin{array}{cc}{\left(\frac{\partial {{\mathcal{P}}}_{1,x}}{\partial t}\right)}^{2} & \frac{\partial {{\mathcal{P}}}_{1,x}}{\partial t}\frac{\partial {{\mathcal{P}}}_{1,y}}{\partial t}\\ \frac{\partial {{\mathcal{P}}}_{1,y}}{\partial t}\frac{\partial {{\mathcal{P}}}_{1,x}}{\partial t} & {\left(\frac{\partial {{\mathcal{P}}}_{1,y}}{\partial t}\right)}^{2}\end{array}\right]$$

where $\overleftrightarrow{I}$ is the identity matrix, ${\boldsymbol{{\mathcal{E}}}}_{1}$ is the pump field, *α* is the pump absorption cross section, and *γ*_1_ and *F*_1_ are the scalar time-varying scattering rate and scalar time-dependant modification to the plasma frequency, both of which drive the pump polarization dynamics ${\boldsymbol{{\mathcal{P}}}}_{1}$. Equations (3) and (4) are directly linked to the more standard dielectric tensor for static materials. From this representation it can be immediately understood that linear pumping leads to a non-scalar diagonal complex matrix imposing both birefringence and dichroism. Conversely, under circular pumping, off-diagonal anti-symmetric terms appear, leading to symmetry breaking. It is worth mentioning that for the case of collinear pumping and probing, the tensor collapses back to a purely scalar problem.

**Fig. 6:**
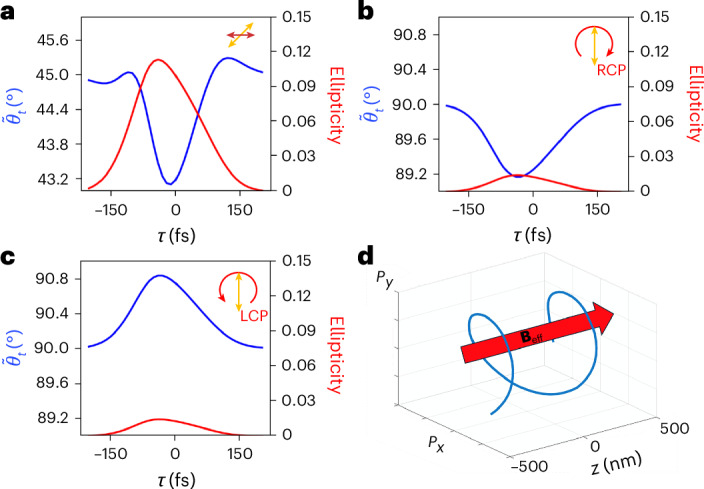
Full-wave simulations with coupled equations and pictorial represention of material polarization under circular pumping. **a**, Polarization rotation angle ${\widetilde{\theta }}_{t}({\omega }_{2})$ (blue line) and ellipticity (red line) as a function of the pump–probe delay *τ* under linearly polarized pumping and probe at 45°. **b**,**c**, same as **a** for left-handed (**b**) and right-handed (**c**) circularly polarized pump beam. All simulations are in close agreement with the reported experimental results in both rotation angle and ellipticity. **d**, Material polarization density vector in the *x* and *y* directions as a function of propagation distance *z*, when the material is driven by a circularly polarized pump. The rotation of the polarization vector induces an effective magnetization **B**_eff_, which breaks time-reversal symmetry and induces non-reciprocity.

Figure 6 reproduces all results attained (both linear and circular pumping) by full-wave simulations using the system described by equation (2). In Fig. 6a the pump wavepacket is linearly polarized along the *x* axis, whereas the probe wavepacket is linearly polarized at 45°, leading to an induced temporal anisotropy and birefringence. Figure 6a also illustrates simulated results for both polarization rotation angle and ellipticity as a function of the pump–probe delay *τ*. Figure 6b,c shows simulation results when the pump beam is LCP and RCP, respectively. Theoretical predictions for both linear and circular pump polarizations show good agreement with the experimental results.

Regarding the induced optical activity, as equations (3) and (4) do not explicitly contain any magnetic field terms, the origin of the symmetry breaking is not immediately apparent. To clarify the underlying mechanism, we can make explicit the effective non-static magnetic field, **B**_eff_, as follows:

5 $${{\bf{B}}}_{\mathrm{eff}}=\frac{{m}_{0}}{e{F}_{1}(z,t)}{\overleftrightarrow{\gamma }}_{2}^{\,\;(a)}(z,t){{\bf{e}}}_{{\rm{z}}}=\frac{{m}_{0}\alpha }{2e}\left({{\mathcal{E}}}_{1,y}\frac{\partial {{\mathcal{P}}}_{1,x}}{{\rm{\partial }}t}-{{\mathcal{E}}}_{1,x}\frac{\partial {{\mathcal{P}}}_{1,y}}{{\rm{\partial }}t}\right){{\bf{e}}}_{{\rm{z}}}$$

6 $${\overleftrightarrow{\gamma }}_{2}^{\;(s)}=\frac{{\overleftrightarrow{\gamma }}_{2}+{\overleftrightarrow{\gamma }}_{2}^{\;T}}{2},\,{\overleftrightarrow{\gamma }}_{2}^{\;(a)}=\frac{{\overleftrightarrow{\gamma }}_{2}-{\overleftrightarrow{\gamma }}_{2}^{\;T}}{2}$$

where *e* is the electron charge, *m*_0_ is the linear effective electron mass, ${{\bf{e}}}_{z}$ is the unit vector orthogonal to the film and ${\overleftrightarrow{\gamma }}_{2}^{\;T}$ is the transpose of ${\overleftrightarrow{\gamma }}_{2}$ (more details on equations (3) and (4) can be found in Supplementary Appendix F). Using the above relations we can rewrite the oscillator equation with respect to an effective magnetization:

7 $$\frac{{{\rm{\partial }}}^{2}{{\boldsymbol{\mathcal{P}}}}_{2}}{{\rm{\partial }}{t}^{2}}+{\overleftrightarrow{\gamma }}_{2}^{\;(s)}\frac{{\rm{\partial }}{{\boldsymbol{\mathcal{P}}}}_{2}}{{\rm{\partial }}t}={\varepsilon }_{0}{\omega }_{{\rm{p}}}^{2}\,{\overleftrightarrow{F}}_{2}(z,t){{\boldsymbol{\mathcal{E}}}}_{2}+\frac{e}{m}\frac{{\rm{\partial }}{{\boldsymbol{\mathcal{P}}}}_{2}}{{\rm{\partial }}t}\times {{\bf{B}}}_{\mathrm{eff}}$$

This formula is functionally identical to equation (2), and it makes explicit how induced nonlinearities, associated with a temporal change in the scattering parameter, can lead to an IFE. This mechanism is also pictorially explained in Fig. 6d, where we show the pump-induced material polarization components ${{\mathcal{P}}}_{x}$ and ${{\mathcal{P}}}_{y}$, as a function of the orthogonal propagation distance **z**. Here, the spiral shape of the polarization vector leads to a non-zero curl current, which is ultimately responsible for the creation of **B**_eff_. This magnetic field allows for breaking time-reversal symmetry and enabling non-reciprocity.

## Conclusions

We have demonstrated an all-optical tunable, ultrafast and reconfigurable platform for controlling the polarization state of light by dynamically inducing birefringence, dichroism and non-reciprocal optical activity in an unstructured, planar, isotropic and sub-wavelength system. Using femtosecond laser excitation upon an AZO thin film within its ENZ wavelength region, we optically induce transient anisotropies in the material’s dielectric response that enable all the three key polarization interaction mechanisms. Notably, this polarization control is achieved without any reliance on resonant structures or static fabrication-induced asymmetry, allowing for broadband operation and extreme temporal agility. Linearly polarized pumping generates transient birefringence and anisotropic absorption, with associated nonlinear phase shifts of up to 0.1π μm^−1^ and induced dichroic ratios of 23%. Circularly polarized pumping introduces dynamically reconfigurable optical activity, with polarization rotation exceeding 1° μm^−1^. These phenomena were quantitatively captured by a first-principles model that attributes the observed effects to ultrafast changes in the electron scattering parameter, which induces symmetry-breaking electron dynamics in the photoexcited plasma.

This work not only establishes a new paradigm for ultrafast polarization modulation via time-varying media but also sets the foundation for a new class of compact and optically programmable photonic devices. The demonstrated compatibility with planar integration and femtosecond response opens up new avenues for applications in ultrafast optical switching, quantum information processing, dynamic metasurfaces and non-reciprocal photonic systems

## Online content

Any methods, additional references, Nature Portfolio reporting summaries, source data, extended data, supplementary information, acknowledgements, peer review information; details of author contributions and competing interests; and statements of data and code availability are available at 10.1038/s41566-026-01886-3.

## Supplementary information

Supplementary Information Supplementary Appendices A–F.

## Data Availability

All data generated in this study are available from the Heriot-Watt University database at 10.17861/c05d69f1-1756-4591-a4db-fa8316da4bfe.
