# Supplementary material for: All-optical polarization control in time-varying low-index films via plasma symmetry breaking
Source: Nat Photonics. 2026 Apr 3;20(6):628–36. doi: 10.1038/s41566-026-01886-3 (PMC13241314; doi:10.1038/s41566-026-01886-3)
Supplement: Supplementary file 1 — Supplementary Appendices A–F. [file 41566_2026_1886_MOESM1_ESM.pdf]

nature photonics

Supplementary information https://doi.org/10.1038/s41566-026-01886-3

All-optical polarization control in time-varying low-index films via plasma symmetry breaking

In the format provided by the authors and unedited

Contents

A Material information **2**
A.1 Dispersion and morphology . . . 2
A.2 Sample fabrication and material details . . . 2

B Thermal effects **3**

C Probe duration **4**

D Experimental methods **4**
D.1 Transfer function recovery . . . 4
D.2 Transient index recovery . . . 6

E Additional results and discussions **6**
E.1 Thin sample comparison . . . 6
E.2 Full experimental results and recovery . . . 7

F Material model **7**

A Material information

A.1 Dispersion and morphology

Scanning electron microscope (SEM) images of the 900 nm AZO sample used in this study are shown in Fig. S1a-d, at various magnifications ranging from 50 µm to 1 µm. Since the surface exhibits only deeply sub-wavelength features, it can be treated as optically flat with respect to the operational wavelength. The electrical and optical properties of oxygen-deprived AZO films grown under these PLD conditions have been thoroughly documented in the earlier materials studies from the same research lineage[10, 5]. For the specific 900 nm-thick film considered in this study Hall measurements and Drude model fits indicate intrinsic free-electron concentrations in the $10^{21}\,\mathrm{cm}^{-3}$ range. Linear dispersion (shown in Fig. S1e) was extracted from reflection and transmission measurements across the 1100–1600 nm range following the methodology outlined in Ref. [10]. Isotropic behaviour of the AZO sample was verified in the linear regime by comparing analyser scans of the transmitted probe in air (blue line) and through the sample (red line) as shown in Fig. 1Sf.

A.2 Sample fabrication and material details

The AZO sample used in this work is the same oxygen-deprived AZO film originally developed within the Boltasseva–Shalaev group [15] and previously characterised in detail by Clerici *et al.* [5]. The film consists of a 900 nm-thick AZO layer deposited on a fused-silica substrate by pulsed laser deposition (PLD). A KrF excimer laser (248 nm) was used to ablate a 2 wt% Al-doped ZnO ceramic target of 99.99 % purity, with a laser fluence of $1.5\,\mathrm{J\,cm}^{-2}$ at the target surface. During deposition, the substrate temperature during deposition was maintained at 75 °C, while the chamber oxygen pressure was held below 10^{-2} mTorr in order to promote oxygen deficiency and enhance the free-electron concentration in the film. These parameters closely follow the established low-oxygen PLD protocol previously used.

The nominal aluminium content is defined by the target composition. Investigations of AZO films grown from ZnO–Al_2O_3 targets in the 1–3 wt% range, consistently report an aluminium concentration of approximately 1–2 at.% in the final films, with Al present in the Al^{3+} oxidation state and substituting Zn sites in the wurtzite lattice. Representative analyses include XPS and RBS studies by Chen *et al.* [3], Chang *et al.* [2], and Horwat *et al.* [8], all of which examined AZO films grown under sputtering or PLD conditions comparable to those used

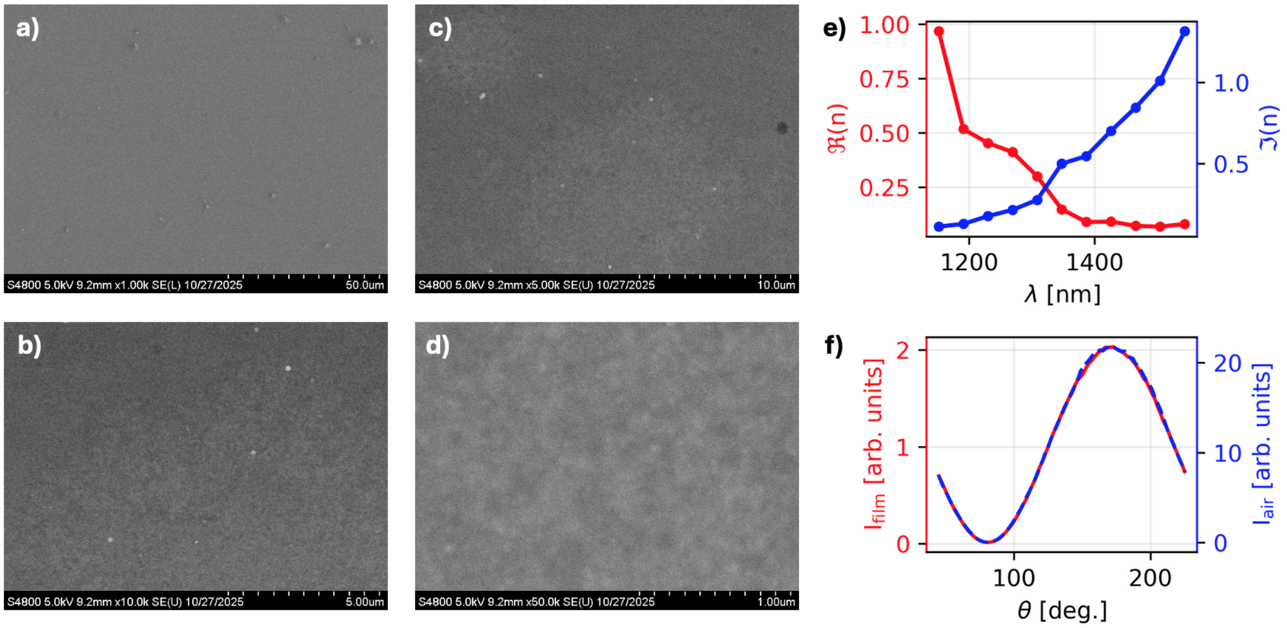

Figure S1: a, b, c, d) Scanning electron microscopy (SEM) images of the oxygen-deprived AZO film recorded at progressively increasing magnifications, with scale bars of 50 µm, 10 µm, 5 µm, and 1 µm, respectively. The surface exhibits only subwavelength features with no long-range order, confirming that any morphological inhomogeneity is deeply subwavelength at the operating wavelength and therefore averaged out by the optical field. e) Linear material dispersion of the AZO film extracted from reflection and transmission measurements and modelled via a transfer-matrix fit [10]. Red line provides real index while blue line provides imaginary index. f) Analyser scan of unpumped probe beam through the AZO film (red line) and through air (blue line). Disregarding the absolute transmitted intensity, both curves overlap with excellent agreement, demonstrating the AZO films isotropic behaviour.

in the present work. These works also confirm that oxygen vacancies introduced during low-oxygen deposition act as additional electron donors. In the case of Horwat *et al.*, RBS measurements fixed the total Al content at 4 at.% and XANES analysis revealed a coexistence of substitutional Al and an Al_2O_3-like octahedral component at higher doping levels. This behaviour is typical of AZO approaching its solid-solubility limit. With the 2 wt% target of the present film, the substitutional fraction is expected to dominate, yielding a homogeneous doped semiconductor.

B Thermal effects

To quantify the temperature rise in the AZO film during optical pumping, we carried out a 2D finite-difference time-domain (FDTD) simulation of heat diffusion including substrate coupling and radiative cooling. The temperature field $T(x, y, t)$ is governed by the heat equation with source and sink terms:

$$\frac{\partial T}{\partial t} = \rho \nabla^2 T + \frac{Q(x, y, t)}{\rho c_p} - \frac{\varepsilon \sigma \left(T^4 - T_{\mathrm{amb}}^4\right)}{\rho c_p} - \frac{h_{\mathrm{sub}}(T - T_{\mathrm{amb}})}{\rho c_p}, \tag{1}$$

where ρ is the thermal diffusivity of the AZO film, ρ is the mass density, c_p is the specific heat capacity, $Q(x, y, t)$ is the absorbed laser power density, ε is the emissivity, σ is the Stefan–Boltzmann constant, T_{amb} is the ambient temperature, and h_{sub} is the effective thermal coupling coefficient describing heat flow into the substrate. The AZO thermal parameters used were: $\rho = 5600\ \mathrm{kg\,m^{-3}}$; $c_p = 500\ \mathrm{J\,kg^{-1}\,K^{-1}}$; $k = 12\ \mathrm{W\,m^{-1}\,K^{-1}}$; $\alpha = k/(\rho c_p) = 4.29 \times 10^{-6}\ \mathrm{m^2\,s^{-1}}$. These values fall within reported ranges for sputtered or PLD-deposited AZO films [13, 16, 1]. Radiative cooling is included using emissivity $\varepsilon = 0.8$, ambient temperature $T_{\mathrm{amb}} = 294$ K, and the Stefan–Boltzmann constant $\sigma = 5.67 \times 10^{-8}\ \mathrm{W\,m^{-2}\,K^{-4}}$. Heat loss to the substrate is modeled with an effective interfacial heat-transfer coefficient of $h_{\mathrm{sub}} = 2 \times 10^4\ \mathrm{W\,m^{-2}\,K^{-1}}$, representative of moderate thin-film thermal boundary conductance. At $t = 0$, we set $T = T_{\mathrm{amb}}$. Laser excitation is modeled as a Gaussian heat source with total optical power $P_{\mathrm{laser}} = 7$ mW, repetition rate 10 Hz, and spot radius of 0.75 mm. The absorbed power density is

$$Q(x, y, t) = AP_{\mathrm{laser}} \exp\left(-\frac{x^2 + y^2}{2r^2}\right) f(t), \tag{2}$$

with $A = 0.3$ assumed absorption and $f(t)$ representing the 10 Hz temporal envelope.

Simulation results (Fig. S2), show a maximum temperature rise of only 17.5 μK after 10 minutes, confirming that the system experiences negligible thermal build up at equilibrium. Consequently, the refractive index

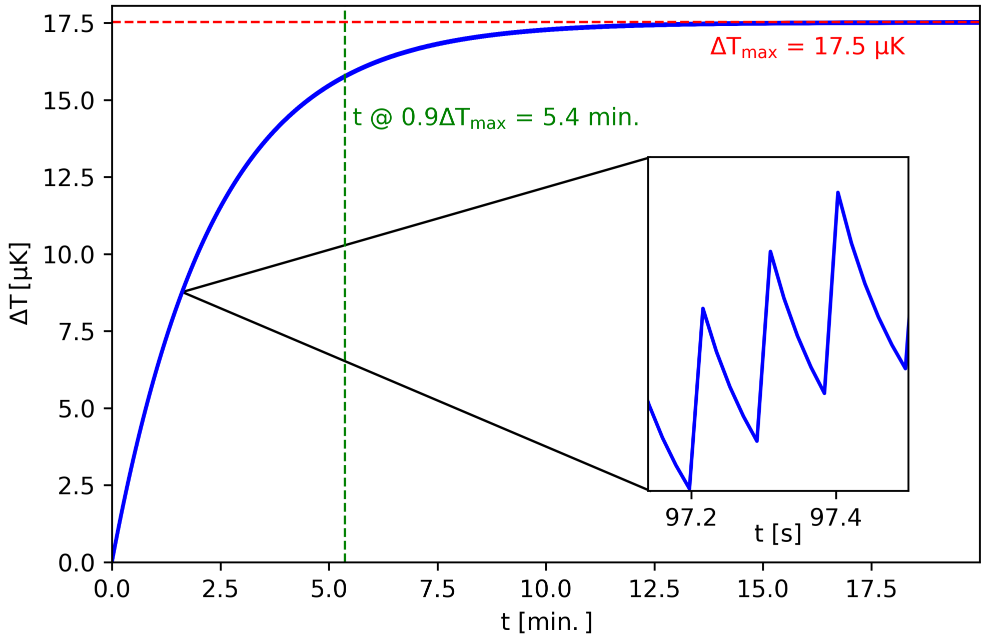

Figure S2: Evolution of the maximum temperature rise (ΔT) in the AZO film as a function of time (in minutes), under 10 Hz, 7 mW pulsed laser excitation. The red dashed line marks the peak temperature ($\mathrm{T_{max}}$), while the green dashed vertical line indicates the time at which the temperature rises to 90% of $\mathrm{T_{max}}$. The inset shows a magnified view of the microsecond-scale, pulse-by-pulse thermal build up.

of AZO remains effectively constant, since thermo-optic shifts require temperature changes many orders of magnitude larger than those observed [11].

C Probe duration

In Fig. S3 we show a frequency resolved optical gating (FROG) measurement of the probe pulse, resulting in an 85 fs pulse duration and a flat spectral phase. The mean RMS error of this retrieval was 4×10^{-4}.

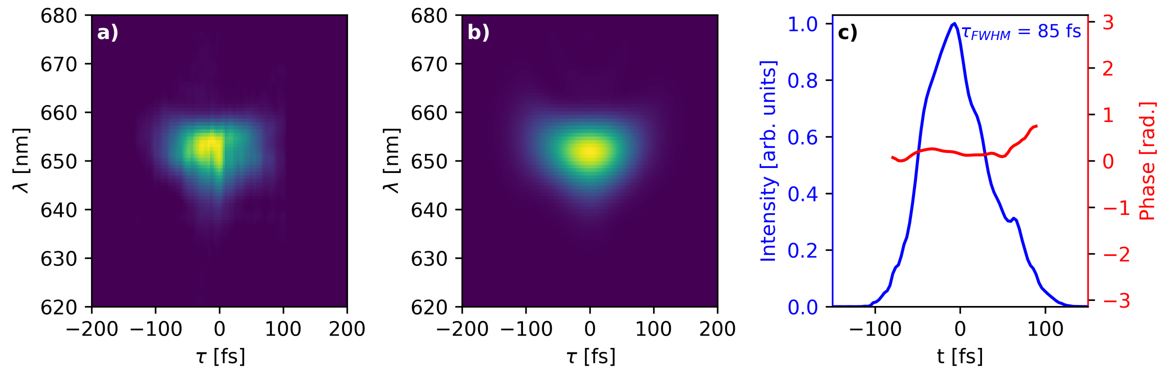

Figure S3: a) Measured frequency–resolved optical gating (FROG) trace of the probe pulse used in the experiment. b) Corresponding reconstructed FROG trace obtained from the retrieval algorithm, showing excellent agreement with the measurement. c) Retrieved temporal amplitude and phase of the probe pulse. The pulse exhibits a full width at half maximum (FWHM) duration of 85 fs and a near-flat spectral phase, confirming the high fidelity and short duration of the probe used for all time-resolved measurements.

D Experimental methods

D.1 Transfer function recovery

The experimentally measured probe intensity at the detector $I_m(\tau, \theta)$, can be written as a function of pump-probe delay τ and polariser angle θ as shown below.

$$I_m(\tau, \theta) = \int_{-\infty}^{\infty} I_T(t, \tau, \theta) dt \tag{3}$$

where $I_T(t, \tau, \theta)$ is the instantaneous transmitted intensity after both the film and polariser at a time t. The time integral over t accounts for the fact that ultrafast pulses are integrated by the power meter. The variable t tracks our probe field evolution, and because of this we set $t = 0$ to be the peak of our probe pulse as extracted from FROG measurements. Since the probe originates from a laser source, and does not propagate through disordered media, we assume it is not unpolarised. Thus, $I_T(t, \tau, \theta)$ can be expressed in terms of the transmitted electric field envelope in the x- and y- directions, and their relative phase difference δ:

$$\begin{aligned} I_T(t, \tau, \theta) = {} & \cos^2(\theta)|E_x(t, \tau)|^2 + \sin^2(\theta)|E_y(t, \tau)|^2 \\ & + 2|E_x(t, \tau)||E_y(t, \tau)| \sin(\theta) \cos(\theta) \cos(\delta(t - \tau)) \end{aligned} \tag{4}$$

This equation is lifted directly from Jones matrix formalism [7], except the constant field amplitude $E_{0,x}$ and $E_{0,y}$, has been replaced with a time varying amplitude as our pump can induce time vayring polarisation dynamics. We define transfer functions $X(t - \tau)$ and $Y(t - \tau)$ to describe the instantaneous field transmission through the film at a pump at a delay of τ.

$$|E_x(t)| = X(t - \tau)|E_{in}(t)| \cos(\varphi_{in}) \tag{5}$$

$$|E_y(t)| = Y(t - \tau)|E_{in}(t)| \sin(\varphi_{in}) \tag{6}$$

Where $|E_{in}(t)|$ is input probe field amplitude and φ_{in} is the probes initial polarisation angle. The probe pulse E_{in} is well characterised, with Gaussian duration 85 fs (FWHM) and central wavelength of 1250 nm, and thus E_{in}. Material dispersion is neglected due to the film's subwavelength thickness, and multiple reflections are ignored given the high absorption. We also do not consider the probes nonlinear contributions, which sets

off-diagonal index terms to zero (see Appendix F section). It is worth noting that in the above definition, when the input field is zero (e.g., outside the pulse duration), we cannot define the value of the transfer function, however, using previous measurements we can set the baseline level of the transfer functions from known linear transmission measurements (Appendix A.1).

Substituting Eqs. 5, 6, into 4, and then into Eq. 3, yields:

$$\begin{aligned} I_m(\tau,\theta) &= \cos^2(\theta)\cos^2(\varphi_{in})\int |E_{in}(t)|^2 X^2(t-\tau)dt \\ &+ \sin^2(\theta)\sin^2(\varphi_{in})\int |E_{in}(t)|^2 Y^2(t-\tau)dt \\ &+ 2\sin(\theta)\cos(\theta)\sin(\varphi_{in})\cos(\varphi_{in})\int |E_{in}(t)|^2 X(t-\tau)Y(t-\tau)\cos(\delta(t-\tau))dt \end{aligned} \tag{7}$$

Fourier transforming gives:

$$\begin{aligned} I_m(\omega,\theta) = \mathcal{F}(I_m(\tau,\theta)) = \mathcal{F}(|E_{in}(t)|^2)[&\cos^2(\theta)\cos^2(\varphi_{in})\mathcal{F}(X^2(t)) \\ &+ \sin^2(\theta)\sin^2(\varphi_{in})\mathcal{F}(Y^2(t)) \\ &+ 2\sin(\theta)\cos(\theta)\sin(\varphi_{in})\cos(\varphi_{in})\mathcal{F}(X(t)Y(t)\cos(\delta(t)))] \end{aligned} \tag{8}$$

Introducing variables $A(\omega)$, $B(\omega)$, and $C(\omega)$:

$$A(\omega) = \mathcal{F}[X^2(t)] \tag{9}$$

$$B(\omega) = \mathcal{F}[Y^2(t)] \tag{10}$$

$$C(\omega) = \mathcal{F}[X(t)Y(t)\cos(\delta(t))] \tag{11}$$

we obtain

$$I_m(\omega,\theta) = \mathcal{F}(|E_{in}(t)|^2)\left[\cos^2(\theta)\cos^2(\varphi_{in})A(\omega) + \sin^2(\theta)\sin^2(\varphi_{in})B(\omega) + 2\sin(\theta)\cos(\theta)\sin(\varphi_{in})\cos(\varphi_{in})C(\omega)\right] \tag{12}$$

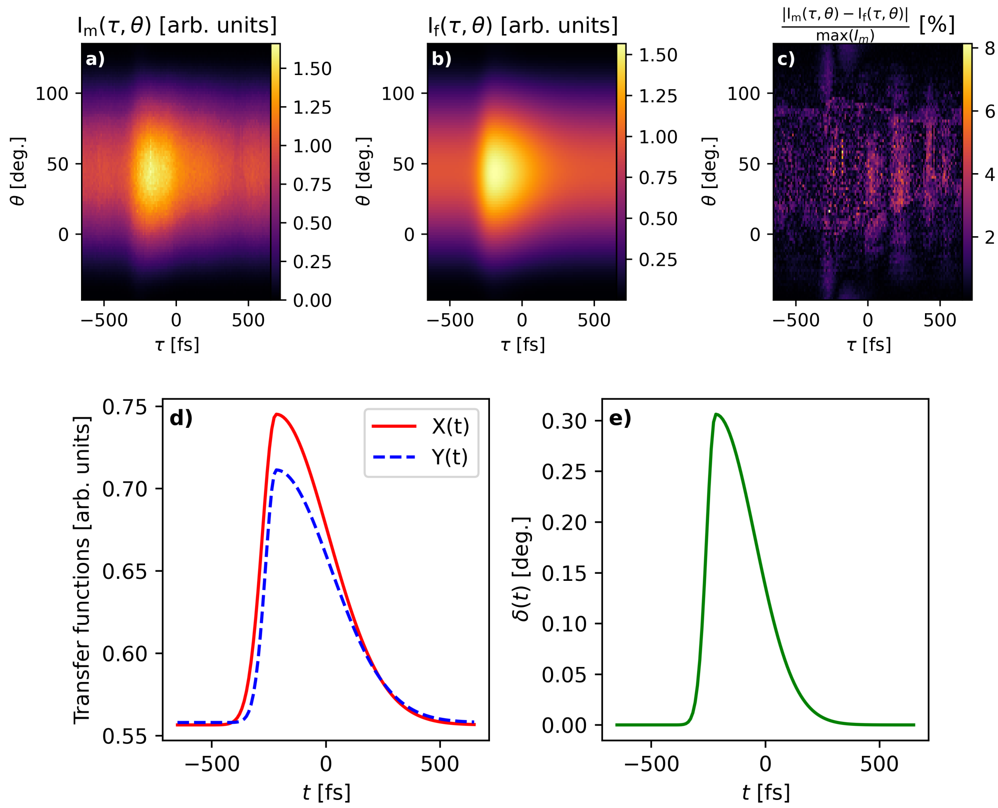

Figure S4: Ultrafast nonlinear polarisation coupling in a time-varying AZO film. a) Experimental intensities against polariser angle and pump-probe delay. b) Fit of experimental data. c) Error of fit to experimental data normalised to the maximum recorded intensity. d) Transient functions used to recover the fit. e) Phase difference between horizontal and vertical electric field used for fit.

Using a global optimisation algorithm (differential evolution [22]), we determine the parameters $X(t)$, $Y(t)$, and $\delta(t)$ that best match our experimental data. Direct fitting is impractical due to the large number of variables, so each function is modelled as a bi-Gaussian with distinct rising and falling times. This choice is justified by prior knowledge of AZO's optical behaviour [5, 20], where nonlinearity stems from hot electron excitation followed by lattice thermalisation, two intrinsically different processes that yield asymmetric responses. Below we show the fitting equations for each of these material responses:

$$X(t) = \left\{ \begin{array}{ll} T_0 + a_x e^{-\frac{t^2}{\tau_{x,1}}}, & \text{if } t < 0 \\ T_0 + a_x e^{-\frac{t^2}{\tau_{x,2}}}, & \text{if } t \geq 0 \end{array} \right\}, \quad Y(t) = \left\{ \begin{array}{ll} T_0 + a_y e^{-\frac{t^2}{\tau_{y,1}}}, & \text{if } t < 0 \\ T_0 + a_y e^{-\frac{t^2}{\tau_{y,2}}}, & \text{if } t \geq 0 \end{array} \right\}, \quad \delta(t) = \left\{ \begin{array}{ll} a_\delta e^{-\frac{t^2}{\tau_{\delta,1}}}, & \text{if } t < 0 \\ a_\delta e^{-\frac{t^2}{\tau_{\delta,2}}}, & \text{if } t \geq 0 \end{array} \right\} \tag{13}$$

where a is the fitted amplitude parameter, τ_1 is the rising time, and τ_2 is the falling time for the functions $X(t)$, $Y(t)$, and $\delta(t)$, as indicated by the subscript. The value T_0 is not a fitting parameter and is instead set by the experimentally measured linear transmission.

The fitted functions are then used to calculate a theoretical fit $I_f(\tau, \theta)$. For the case of a 45° polarised probe the experimental data and the error is shown in Fig. S4a, b, and c, respectively. We also plot the film transfer functions $X(t)$, $Y(t)$, and, $\delta(t)$, in Fig S4c and d. These functions can be used to calculate $E_x(t)$ and $E_y(t)$ for any pump-probe delay. For clarity the manuscript only reports the average polarisation state of the transmitted pulse at a given pump-probe delay. Finally, we define the ellipticity used within the main manuscript as [7, 6]

$$\epsilon = \tan\left(\frac{1}{2}\sin^{-1}\left(\frac{2|E_x||E_y|\sin\delta}{|E_x|^2 + |E_y|^2}\right)\right) \tag{14}$$

D.2 Transient index recovery

At normal incidence, the transfer function through a lossy thin film can be written as.

$$X(t) = \left|\sqrt{T_{air\to AZO} P_{AZO} T_{AZO\to SiO_2} T_{SiO_2\to air}}\right| \tag{15}$$

Where $T_{A\to B}$ describes the transmission through an interface between materials a and b. P_A describes propagation through material A (where propagation through the silica substrate has been neglected due to negligible losses and a flat phase contribution in time for both $E_x(t)$ and $E_y(t)$). Using Fresnel equations we can expand as follows:

$$X(t) = \left|\sqrt{\left(1 - \left|\frac{1 - n_x(t)}{1 + n_x(t)}\right|^2\right) \left|e^{-4\pi i \frac{d}{\lambda_c} \operatorname{Im}(n_x(t))}\right| \left(1 - \left|\frac{n_x(t) - n_s}{n_x(t) + n_s}\right|^2\right) T_{SiO_2\to air}}\right| \tag{16}$$

Where $\lambda_c = 2\pi c/\omega_c$, d is the film thickness, and n_s is the substrate index (which is taken to be 1.45 for silica glass), and $n_x(t)$ is the time varying film index in the x direction. The term $T_{SiO_2\to air}$ is the temporally static scalar Fresnel coefficient for transmission from SiO_2 to air, and is evaluated to be ≈ 0.97. An analogous equation can be written for $Y(t)$. Using these equations alongside our knowledge of the material dispersion, which fixes the nonlinear change in n_i versus n_r, we can recover the complex transient index of the AZO film.

E Additional results and discussions

E.1 Thin sample comparison

In Fig. S5a we provide the raw experimental data for the polarisation control experiment on the 300 nm AZO sample. Experimental conditions were kept identical to those used in the main manuscript, specifically, the pump was set to 800 GW/cm^2 at 787 nm, and the probe was set at 1250 nm. Then, at peak temporal overlap (maximum nonlinearity), the transmitted intensity was measured as a function of analyser angle. As with the 900 nm sample, the total transmitted intensity increases under pumping, however no ellipticity is imparted due to the reduced propagation distance (e.g., the minimised transmission does not increase when pumped). For comparison, results from the 900 nm sample are also shown in Fig. S5b. A clear increase to the ellipticity is observed. In contrast, the 300 nm pumped sample still imparts a small polarisation angle shift of 0.25 degrees, as compared to the 1.5° of rotation measured in the 900 nm sample.

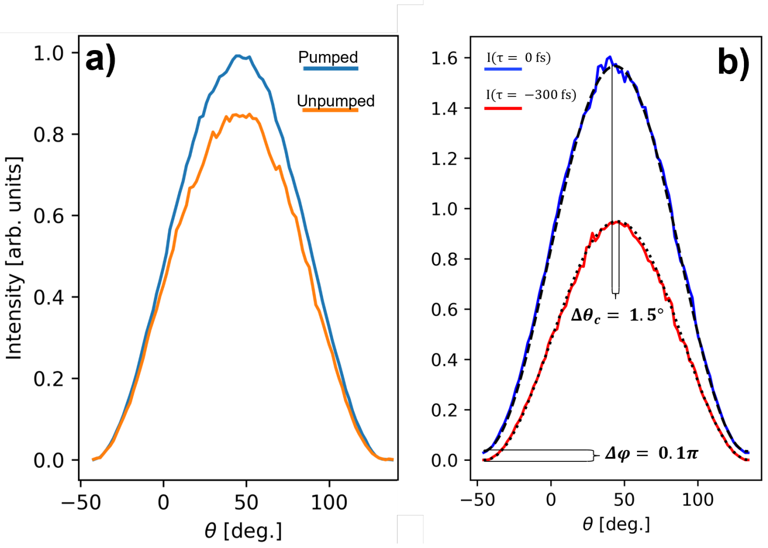

Figure S5: a) Transmitted intensity through a 300 nm AZO sample against analyser angle. Orange line provides unpumped case while the blue line shows pumped case (800 GW/cm^2 pump at 787 nm, with probe at 1250 nm). b) Data slices from Fig. S3a in the main manuscript, provided for comparative purposes. The ellipticity (increase of the minimum transmission when pumped) and the polarisation rotation (shift of the entire curve) have been highlighted.

E.2 Full experimental results and recovery

Figures S6 and S7 presents complete experimental results and corresponding fits for orthogonal and parallel pumping, and for left handed and right handed pumping, respectively (see indicated icons). The maximum values shown in colorbar of Fig. S6a and Fig.S6c highlight that the relative nonlinear response under orthogonal pumping is significantly weaker than that observed with parallel pumping.

F Material model

The linear optical response of TCOs such as indium tin oxide (ITO) and AZO is well described by the Drude model, particularly near the epsilon-near-zero (ENZ) crossing point. However, at high optical intensities, on the order of few TW/cm^2, nonlinear contributions dominate the light-matter interactions exposing the limitations of the standard Drude model [4, 9, 21, 20]. For our experimental settings we operate far from any Lorentzian resonance and consequently, multiphoton nonlinearities are negligible. Nonlocal effects can also be discounted because they are effective for layers that are only tens of nanometres in thickness. Extending the Drude model into the nonlinear regime is nontival, and several approaches have been proposed to address them. Here, we focus on the key contributions to nonlinearity that describe the pump-probe dynamics occurring across a broad frequency range around the ENZ frequency. The primary source of nonlinearity is the generation of hot electrons. When a TCO is pumped near the ENZ crossover point, conduction band electrons are heated, resulting in a modification of their effective mass [18]. It is worth pointing out that in our model we do not restrain ourselves to third order nonlinearities, but we rather consider a coupled hot-electron system which intrinsically accounts for high order terms. More precisely, the effective mass term could be expanded to explicitly separate higher-order nonlinear contributions. We interpret the hot-electron effective mass change as an intrinsically time dependant process [20]. Accordingly, we define $m(\mathbf{r},t) = m_0[1+\delta_m(\mathbf{r},t)]$, and we model the change in the effective mass $\delta_m(\mathbf{r},t)$ using the following equation

$$\frac{\partial \delta_m(\mathbf{r},t)}{\partial t} + \gamma_m \delta_m(\mathbf{r},t) = \alpha \frac{\partial \boldsymbol{\mathcal{P}}(\mathbf{r},t)}{\partial t} \cdot \boldsymbol{\mathcal{E}}(\mathbf{r},t), \tag{17}$$

Here, $\boldsymbol{\mathcal{P}}(\mathbf{r},t)$ is the polarization vector associated to the conduction band electrons and $\boldsymbol{\mathcal{E}}(\mathbf{r},t)$ is the electric field. The parameters α and γ_m correspond to the efficiency of energy transfer from the pump and the relaxation rate of the medium back to its original state, respectively. Taking into account that the effective mass m is now a function of time, the Drude model must be modified accordingly. As pointed out in [20], in optical contexts the effective mass of free carriers is usually assumed to be constant. Since the effective mass is now explicitly time dependent, the standard Drude model is replaced by a hydrodynamic-Maxwell approach that accounts for this variation, leading to the appearance of an additional damping term proportional to the rate of change of

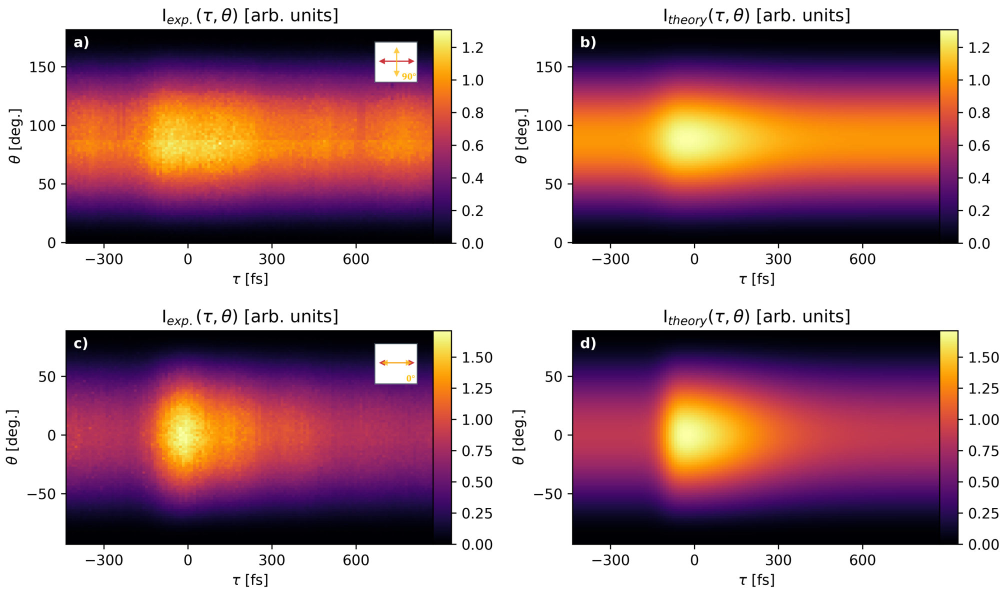

Figure S6: Full experimental data and fits for and orthogonal and parallel pumping scheme (see icons). a) Experimentally measured intensity map for a vertically polarised probe. b) Fitted intensity distribution for vertically polarised probe. c) Extracted transient complex index in the y direction (orthogonal to the pump). d) Experimentally measured intensity map for a horizontally polarised probe.

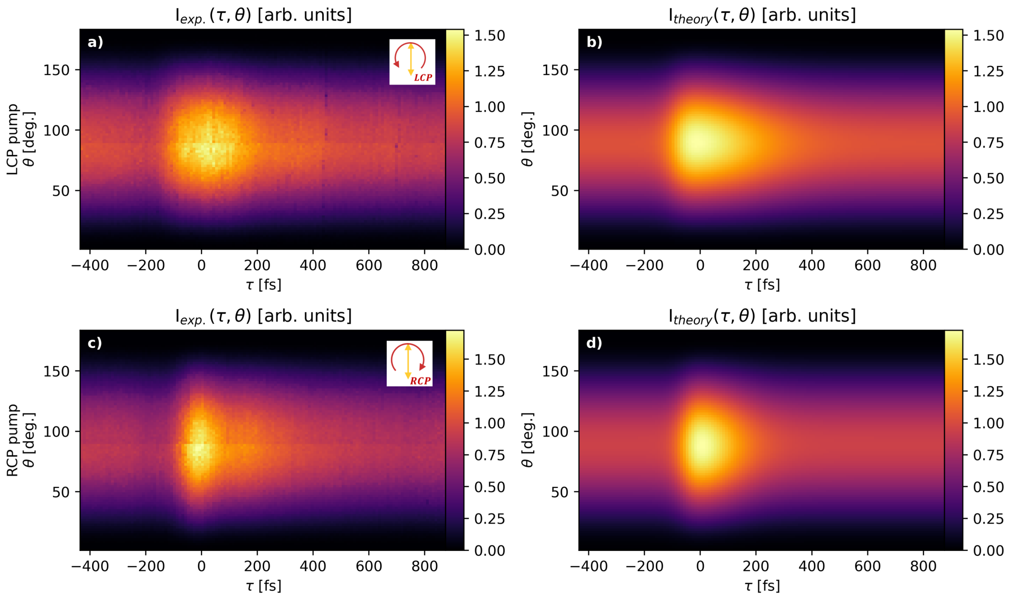

Figure S7: Full experimental data and fits for and left handed and right handed pumping cases (see icons). a) Measured probe transmission as a function of analyser angle θ and pump–probe delay τ for a left-handed circularly polarised (LCP) pump. b) Theoretical fit to the probe's measured intensity distribution for a LCP pump. c) Measured probe transmission for a right-handed circularly polarised (RCP) pump. d) Theoretical fit to the probe's measured intensity distribution for a RCP pump.

the effective mass with respect to time. Thus, the temporal dynamics of the polarization vector is governed by the second-order differential equation,

$$\frac{\partial^2 \boldsymbol{\mathcal{P}}}{\partial t^2} + \left(\gamma + \frac{1}{1+\delta_m}\frac{\partial \delta_m}{\partial t}\right)\frac{\partial \boldsymbol{\mathcal{P}}}{\partial t} = \varepsilon_0 \frac{\omega_p^2}{1+\delta_m}\boldsymbol{\mathcal{E}}. \tag{18}$$

where γ, ω_p are the standard scattering rate of electrons and plasma frequency, respectively (ϵ_0 is the vacuum permittivity). The term proportional to $\frac{\partial \delta_m}{\partial t}$ originates directly from $\frac{1}{m(r,t)}\frac{\partial m(r,t)}{\partial t}$, imposed by the temporal dependence of the effective mass [20]. This additional scattering-rate rate contribution represents a novel extension of the Drude framework into the nonlinear, time-dependent regime. Unlike conventional treatments, where scattering is assumed static, the explicit incorporation of the effective mass's temporal variation provides a new mechanism for describing ultrafast electron dynamics. This innovation plays a pivotal role in governing the behaviour of polarized wave packets, directly determining the induced anisotropy and enabling the emergence of magneto-optical chiral effects in otherwise isotropic media.

To validate our experimental findings independently, we perform full-wave numerical simulations. More precisely, we consider wave-packets normally incident on the AZO film on the substrate of SiO_2, where the electric field is given by $\boldsymbol{\mathcal{E}}(z,t) = \mathcal{E}_x(z,t)\hat{\mathbf{e}}_x + \mathcal{E}_y(z,t)\hat{\mathbf{e}}_y$. Here and throughout, $\hat{\mathbf{e}}_\alpha$ denotes the unit vector in the α-direction, with $\alpha = x, y, z$.

The incident field is given by $\boldsymbol{\mathcal{E}}_{in}(t) = Re[\mathbf{E}_{in,1}(t) + \mathbf{E}_{in,2}(t)]$, where $\mathbf{E}_{in,j}(t) = A_j e^{i\omega_j(t-t_j)-(t-t_j)^2/s_j^2}\hat{\mathbf{e}}_j$ with $\hat{\mathbf{e}}_j = \cos\theta_j \hat{\mathbf{e}}_x + e^{i\delta_j}\sin\theta_j \hat{\mathbf{e}}_y$ $(j = 1, 2)$. The amplitudes A_j are set to achieve an incident pump intensity of 800 GW/cm^2 and an incident probe intensity of 0.8 GW/cm^2. We set s_1 = 85.0 fs and s_2 = 72.2 fs and the carrier frequencies are $\omega_1 = 2.39 \cdot 10^{15}$ Hz, $\omega_2 = 1.51 \cdot 10^{15}$ Hz (corresponding to λ_1 = 787 nm, λ_2 = 1250 nm ,respectively). The times t_1 and t_2 are chosen to effectively excite the polarized wave packets at the numerical boundary and to get the desired pump-probe delay (i.e. $\tau = t_2 - t_1$). The AZO film (with a thickness 900 nm)is sandwiched between two semi-infinite regions filled with air and SiO_2, respectively. In these two regions, we neglect temporal dispersion and nonlinear effects and, consequently, we suppose that the electric field evolves according to the standard wave equation where light velocity is c and c/n_{SiO_2} in the air and substrate, respectively. Within the AZO slab, the field dynamics is governed by a modified wave equation that accounts for the coupling between the electromagnetic wave and AZO. This modified equation is expressed as follows

$$\frac{1}{c_b^2}\frac{\partial^2 \boldsymbol{\mathcal{E}}(z,t)}{\partial t^2} - \frac{\partial^2 \boldsymbol{\mathcal{E}}(z,t)}{\partial z^2} = -\mu_0 \frac{\partial^2 \boldsymbol{\mathcal{P}}(z,t)}{\partial t^2}, \tag{19}$$

where $c_b = c/\sqrt{\epsilon_b}$ with ϵ_b is a background dielectric permittivity that can effectively account for the contributions from bound electrons, interband transitions, and lattice effects (here, μ_0 is the vacuum permeability). We solve Equation (19) along with Equation (17) and Equation (18) using the PDE module in COMSOL Multiphysics, version 6.3 (COMSOL AB, Stockholm, Sweden). Standard scattering boundary conditions are applied at both the input and output interfaces of the computational domain. In all simulations, we set $\gamma = 8.76 \cdot 10^{13}$ Hz, $\omega_p = 2.54 \cdot 10^{15}$ Hz, $\epsilon_b = 2.96$, $\alpha = 4 \cdot 10^{-9}$ m^2/N, $\gamma_m = 1.14 \cdot 10^{13}$ Hz

In a pump–probe configuration, the total electric field can be written as $\boldsymbol{\mathcal{E}}(z,t) = \boldsymbol{\mathcal{E}}_1(z,t), + \boldsymbol{\mathcal{E}}_2(z,t)$, where the subscripts 1 and 2 denote the pump and probe fields, respectively. In the regime where the probe intensity is much lower than that of the pump, the probe field experiences a linear response. The dynamics of the pump is given by

$$\frac{\partial^2 \boldsymbol{\mathcal{P}}_1}{\partial t^2} + \gamma_1(z,t)\frac{\partial \boldsymbol{\mathcal{P}}_1}{\partial t} = \varepsilon_0 \omega_p^2 F_1(z,t)\boldsymbol{\mathcal{E}}_1, \tag{20}$$

with $\delta_m \simeq \alpha e^{-\gamma_m t}\int_0^t dt' e^{\gamma_m t'} \frac{\partial \boldsymbol{\mathcal{P}}_1}{\partial t'} \cdot \boldsymbol{\mathcal{E}}_1$, and where we define $\gamma_1 = \gamma - \gamma_m F_1 \delta_m + \alpha F_1 \frac{\partial \boldsymbol{\mathcal{P}}_1}{\partial t} \cdot \boldsymbol{\mathcal{E}}_1$, $F_1 = 1/(1+\delta_m)$. On the other hand, by maintaining the leading terms, the light-matter interaction of the probe is described by the following equation.

$$\frac{\partial^2 \boldsymbol{\mathcal{P}}_2}{\partial t^2} + \overleftrightarrow{\gamma}_2(t,z)\frac{\partial \boldsymbol{\mathcal{P}}_2}{\partial t} = \varepsilon_0 \omega_p^2 \overleftrightarrow{F}_2(t,z)\boldsymbol{\mathcal{E}}_2, \tag{21}$$

where

$$\overleftrightarrow{\gamma}_2 = \gamma_1 \overleftrightarrow{I} + \alpha F_1 \begin{bmatrix} \mathcal{E}_{1,x}\frac{\partial \mathcal{P}_{1,x}}{\partial t} & \mathcal{E}_{1,y}\frac{\partial \mathcal{P}_{1,x}}{\partial t} \\ \mathcal{E}_{1,x}\frac{\partial \mathcal{P}_{1,y}}{\partial t} & \mathcal{E}_{1,y}\frac{\partial \mathcal{P}_{1,y}}{\partial t} \end{bmatrix} \tag{22}$$

$$\overleftrightarrow{F}_2 = F_1 \overleftrightarrow{I} - \frac{\alpha F_1}{\epsilon_0 \omega_p^2} \begin{bmatrix} \left(\frac{\partial \mathcal{P}_{1,x}}{\partial t}\right)^2 & \frac{\partial \mathcal{P}_{1,x}}{\partial t}\frac{\partial \mathcal{P}_{1,y}}{\partial t} \\ \frac{\partial \mathcal{P}_{1,y}}{\partial t}\frac{\partial \mathcal{P}_{1,x}}{\partial t} & \left(\frac{\partial \mathcal{P}_{1,y}}{\partial t}\right)^2 \end{bmatrix} \tag{23}$$

with $\overleftrightarrow{I}$ is the identity matrix. Equation (21) establishes a general framework for time-varying non-reciprocal magneto-optical coupling resulting from the pump interaction. Both $\overleftrightarrow{\gamma}_2$ and $\overleftrightarrow{F}_2$ are complete tensors whose

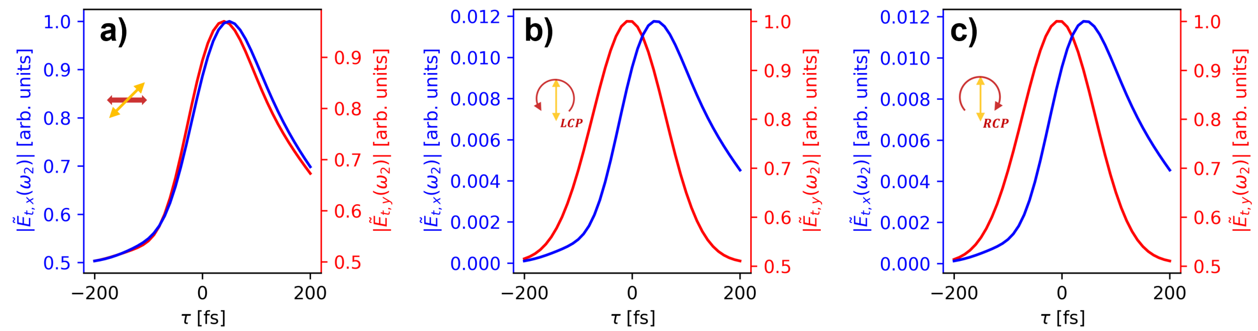

Figure S8: Field amplitudes from full-wave simulations with coupled equations (as shown in main manuscript Fig. S6. The spectral x, and y components of the transmitted electric fields at frequency ω_2 are shown in panel a) as functions of the delay time τ. This is for the case where the pump wave packet is linearly polarized along the x-axis (i.e., $\theta_1 = 0°$ and $\delta_1 = 0°$), and the probe wave packet is linearly polarized with $\theta_2 = 45°$ and $\delta_2 = 0°$. b) Field amplitude from simulation where the pump beam is left circularly polarized with $\theta_1 = 45°$ and $\delta_1 = 90°$, while the probe beam being linearly polarized along the y-axis (i.e., $\theta_2 = 90°$ and $\delta_2 = 0°$). c) Field amplitude from simulation where the pump beam is right circularly polarized with $\theta_1 = 45°$ and $\delta_1 = -90°$, while the probe beam being linearly polarized along the y-axis (i.e., $\theta_2 = 90°$ and $\delta_2 = 0°$).

components vary with the pump field, which in turn depends on time and space. By adjusting the pump parameters, one can, in principle, control both the magnitude and nature of light–matter interaction symmetry. Under circularly polarized pump, the probe experiences a non-reciprocal magneto-optical coupling. This response arises from the antisymmetric component of γ_2 ($\gamma_2^{(a)}$) or equivalently from the effective magnetic field B_{eff}. Herein lies the novelty of our combined experimental and theoretical results. Importantly, we note that Eq. (21) holds in the single-cycle regime [14, 20, 12, 17, 19, 23].

Additional simulations results are provided in Fig. S8, where we show the field amplitudes versus pump-probe delay for the cases of a 45-degree pump-probe configuration (Fig. S8a), a left circularly polarised pump (Fig. S8b), and a right circularly polarised pump (Fig. S8c), as depicted in the icons. Notably, the field amplitudes for the latter two cases are identical, the only distinction being the opposite sign of the $\tilde{E}_x$ component between b) and c). In the main manuscript the effect of polarization is evaluated in the spectral domain, where $\tilde{E}_x$ and $\tilde{E}_y$ are the spectral components of the electric field. At this point we can define the polarization ratio as $\tilde{E}_y/\tilde{E}_x = tan(\tilde{\theta}_t)\exp^{(i\tilde{\delta}_t)}$ where $\tilde{\theta}_t$ and $\tilde{\delta}_t$ are the spectral polarization angle and phase delay, respectively. From this, we estimate the polarisation rotation angle and ellipticity for the output probe as shown in Fig. S6a, b and c.

References

[1] T. El-Brolossy, O. Saber, and S. Ibrahim. "Determining the thermophysical properties of Al-doped ZnO nanoparticles by the photoacoustic technique". In: *Chinese Physics B* 22.7 (2013), p. 074401.

[2] J. Chang, W. Lin, and M.-H. Hon. "Effects of post-annealing on the structure and properties of Al-doped zinc oxide films". In: *Applied Surface Science* 183.1-2 (2001), pp. 18–25.

[3] M. Chen, X. Wang, Y. Yu, Z. Pei, X. Bai, C. Sun, R. Huang, and L. Wen. "X-ray photoelectron spectroscopy and auger electron spectroscopy studies of Al-doped ZnO films". In: *Applied Surface Science* 158.1-2 (2000), pp. 134–140.

[4] A. Ciattoni, C. Rizza, A. Marini, A. D. Falco, D. Faccio, and M. Scalora. "Enhanced nonlinear effects in pulse propagation through epsilon-near-zero media". In: *Laser & Photonics Reviews* 10.3 (2016), pp. 517–525.

[5] M. Clerici, N. Kinsey, C. DeVault, J. Kim, E. G. Carnemolla, L. Caspani, A. Shaltout, D. Faccio, V. Shalaev, A. Boltasseva, et al. "Controlling hybrid nonlinearities in transparent conducting oxides via two-colour excitation". In: *Nature communications* 8.1 (2017), p. 15829.

[6] E. Collett. *Field guide to polarization*. Vol. 15. SPIE press Bellingham, 2005.

[7] G. R. Fowles. *Introduction to modern optics*. Courier Corporation, 2012.

[8] D. Horwat, M. Jullien, F. Capon, J.-F. Pierson, J. Andersson, and J. L. Endrino. "On the deactivation of the dopant and electronic structure in reactively sputtered transparent Al-doped ZnO thin films". In: *Journal of Physics D: Applied Physics* 43.13 (2010), p. 132003.

[9] W. Jaffray, F. Belli, S. Stengel, M. A. Vincenti, M. Scalora, M. Clerici, V. M. Shalaev, A. Boltasseva, and M. Ferrera. “High-order nonlinear frequency conversion in transparent conducting oxide thin films”. In: *Advanced Optical Materials* 12.28 (2024), p. 2401249.

[10] W. Jaffray, M. Clerici, B. Heijnen, A. Boltasseva, V. M. Shalaev, and M. Ferrera. “Nonlinear Loss Engineering in Near-Zero-Index Bulk Materials”. In: *Advanced Optical Materials* 12.1 (2024), p. 2301232.

[11] J. Kim, S. Shrestha, M. Souri, J. G. Connell, S. Park, and A. Seo. “High-temperature optical properties of indium tin oxide thin-films”. In: *Scientific reports* 10.1 (2020), p. 12486.

[12] H. Li, S. Yin, and A. Alù. “Nonreciprocity and Faraday rotation at time interfaces”. In: *Physical Review Letters* 128.17 (2022), p. 173901.

[13] C. P. Liu and G. R. Jeng. “Properties of aluminum doped zinc oxide materials and sputtering thin films”. In: *Journal of Alloys and Compounds* 468.1-2 (2009), pp. 343–349.

[14] E. Lustig, O. Segal, S. Saha, E. Bordo, S. N. Chowdhury, Y. Sharabi, A. Fleischer, A. Boltasseva, O. Cohen, V. M. Shalaev, et al. “Time-refraction optics with single cycle modulation”. In: *Nanophotonics* 12.12 (2023), pp. 2221–2230.

[15] G. V. Naik, V. M. Shalaev, and A. Boltasseva. “Alternative plasmonic materials: beyond gold and silver”. In: *Advanced materials* 25.24 (2013), pp. 3264–3294.

[16] N. Oka, K. Kimura, T. Yagi, N. Taketoshi, T. Baba, and Y. Shigesato. “Thermophysical and electrical properties of Al-doped ZnO films”. In: *Journal of Applied Physics* 111.9 (2012).

[17] V. Pacheco-Peña and N. Engheta. “Temporal aiming”. In: *Light: Science & Applications* 9.1 (2020), p. 129.

[18] D. M. Riffe. “Temperature dependence of silicon carrier effective masses with application to femtosecond reflectivity measurements”. In: *Journal of the Optical Society of America B* 19.5 (2002), pp. 1092–1100.

[19] C. Rizza, G. Castaldi, and V. Galdi. “Spin-controlled photonics via temporal anisotropy”. In: *Nanophotonics* 12.14 (2023), pp. 2891–2904.

[20] M. Scalora, M. Vincenti, D. de Ceglia, N. Akozbek, M. Ferrera, C. Rizza, A. Alù, N. Litchinitser, C. Cojocaru, and J. Trull. “Extreme electrodynamics in time-varying media”. In: *Physical Review A* 112.1 (2025), p. 013502.

[21] M. Scalora, J. Trull, D. de Ceglia, M. A. Vincenti, N. Akozbek, Z. Coppens, L. Rodríguez-Suné, and C. Cojocaru. “Electrodynamics of conductive oxides: Intensity-dependent anisotropy, reconstruction of the effective dielectric constant, and harmonic generation”. In: *Physical Review A* 101.5 (2020), p. 053828.

[22] R. Storn and K. Price. “Differential evolution–a simple and efficient heuristic for global optimization over continuous spaces”. In: *Journal of global optimization* 11.4 (1997), pp. 341–359.

[23] S. Yin, Y.-T. Wang, and A. Alù. “Temporal optical activity and chiral time-interfaces”. In: *Optics Express* 30.26 (2022), pp. 47933–47941.
